# Cell-Free miRNAs as Non-Invasive Biomarkers in Brain Tumors

**DOI:** 10.3390/diagnostics13182888

**Published:** 2023-09-08

**Authors:** Ozal Beylerli, Manuel de Jesus Encarnacion Ramirez, Alina Shumadalova, Tatiana Ilyasova, Mikhail Zemlyanskiy, Aferin Beilerli, Nicola Montemurro

**Affiliations:** 1 Bashkir State Medical University, 450008 Ufa, Russia; 2 Department of Neurosurgery, Peoples’ Friendship University of Russia (RUDN University), 117198 Moscow, Russia; 3 Department of Neurosurgery, Podolsk Regional Hospital, 141110 Moscow, Russia; 4 Department of Obstetrics and Gynecology, Tyumen State Medical University, 625000 Tyumen, Russia; 5 Department of Neurosurgery, Azienda Ospedaliero Universitaria Pisana (AOUP), University of Pisa, 56100 Pisa, Italy

**Keywords:** cell-free microRNAs, blood, cerebrospinal fluid, expression, biomarkers, brain tumors, diagnosis

## Abstract

Diagnosing brain tumors, especially malignant variants, such as glioblastoma, medulloblastoma, or brain metastasis, presents a considerable obstacle, while current treatment methods often yield unsatisfactory results. The monitoring of individuals with brain neoplasms becomes burdensome due to the intricate tumor nature and associated risks of tissue biopsies, compounded by the restricted accuracy and sensitivity of presently available non-invasive diagnostic techniques. The uncertainties surrounding diagnosis and the tumor’s reaction to treatment can lead to delays in critical determinations that profoundly influence the prognosis of the disease. Consequently, there exists a pressing necessity to formulate and validate dependable, minimally invasive biomarkers that can effectively diagnose and predict brain tumors. Cell-free microRNAs (miRNAs), which remain stable and detectable in human bodily fluids, such as blood and cerebrospinal fluid (CSF), have emerged as potential indicators for a range of ailments, brain tumors included. Numerous investigations have showcased the viability of profiling cell-free miRNA expression in both CSF and blood samples obtained from patients with brain tumors. Distinct miRNAs demonstrate varying expression patterns within CSF and blood. While cell-free microRNAs in the blood exhibit potential in diagnosing, prognosticating, and monitoring treatment across diverse tumor types, they fall short in effectively diagnosing brain tumors. Conversely, the cell-free miRNA profile within CSF demonstrates high potential in delivering precise and specific evaluations of brain tumors.

## 1. Introduction

Primary brain tumors, occurring at a rate of approximately 22 cases per 100,000 people, contribute to less than 2% of all newly diagnosed growths. However, about 33% of these primary brain tumors are of a malignant nature, accompanied by a projected 5-year survival rate of 34.4% [1]. Patient outcomes and survival hinge on the specific tumor type. The most promising forecast is linked to pilocytic astrocytoma (with a 5-year survival rate reaching 94.2%), succeeded by meningioma (65.2%) and central nervous system (CNS) lymphoma (29.2%). Conversely, patients grappling with glioblastoma (classified as WHO grade IV) face a considerably lower 5-year survival rate of merely 5.1% [1]. Furthermore, metastases to the brain originating from malignancies elsewhere constitute another substantial cluster of tumors, and despite typically modest survival rates for patients with brain-invading metastases, the prognostic outlook varies individually [2].

The role of diagnosis in predicting outcomes and guiding optimal treatment selection for brain tumors is of paramount importance. Despite significant recent progress in diagnosing brain tumors using diverse modifications of visualization methods followed by subsequent histopathological analysis, the identification of tumors still faces limitations related to tumor size, location, and the heterogeneous composition of their tissue [3]. As a result, the development of innovative diagnostic strategies is necessary, ones that can synergize with existing techniques to enhance diagnostic precision. A highly promising approach for diagnosing a multitude of tumors is liquid biopsy, which involves the identification and quantification of various cell-free biomolecules in human body fluids, such as blood or cerebrospinal fluid (CSF) (Table 1) [4,5,6,7,8].

**Figure 1:**
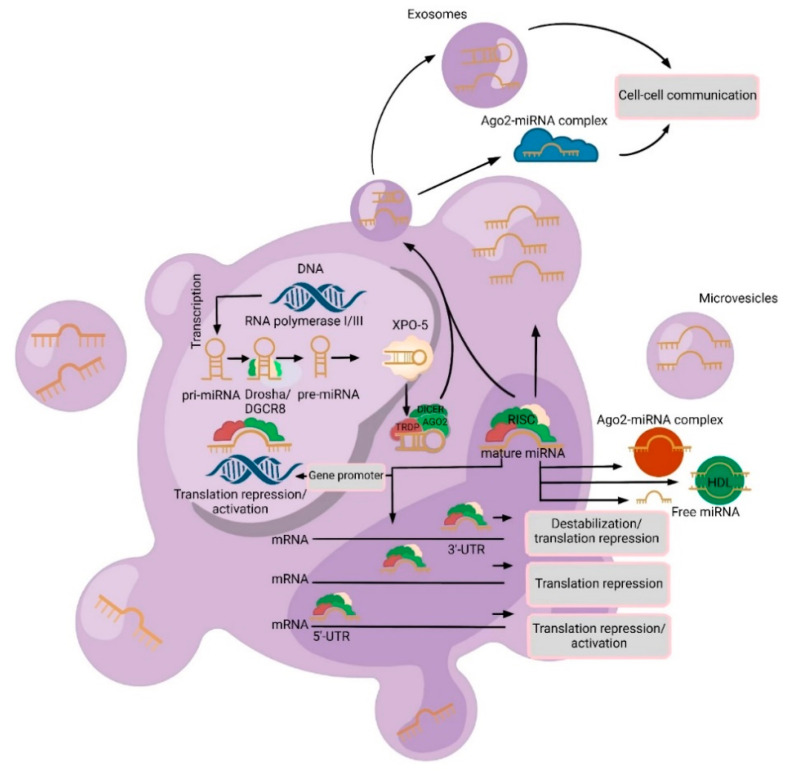
A schematic biogenesis pathway, function, and mechanisms of secretion of microRNA (miRNA). MiRNA biogenesis begins in the nucleus where miRNA genes are transcribed as primary miRNAs (pri-miRNAs) by RNA polymerase II/III. Pri-miRNAs are processed by the RNase III endonuclease and Drosha/DGCR8 complex into precursor miRNAs (pre-miRNA). Then, these pre-miRNAs are exported to the cytoplasm by Exportin 5 (XPO5) and further processed by DICER, a Ribonuclease III enzyme that produces the mature miRNA. Mature miRNAs are incorporated into an RNA-induced silencing complex (RISC), which contains DICER and Argonaute 2 (Ago2) proteins, to produce target mRNA degradation/translational repression/activation. MiRNAs can be secreted from cells into biofluids via the following mechanisms: (1) via extracellular vesicles (EVs) (exosomes or microvesicles (MVs)); (2) as an Ago2-miRNA complex (which represents 90% of cell-free miRNAs) and in the high-density lipoprotein (HDL) complex; and (3) passive secretion through apoptosis or necrosis (free miRNAs).

MicroRNAs (miRNAs) are short, consisting of 18 to 22 nucleotides, single-stranded non-coding RNA molecules that influence gene activity by attaching to specific sites within the 3′ untranslated region (3′ UTR) of target messenger RNAs (mRNAs) (Figure 1).

**Figure 2:**
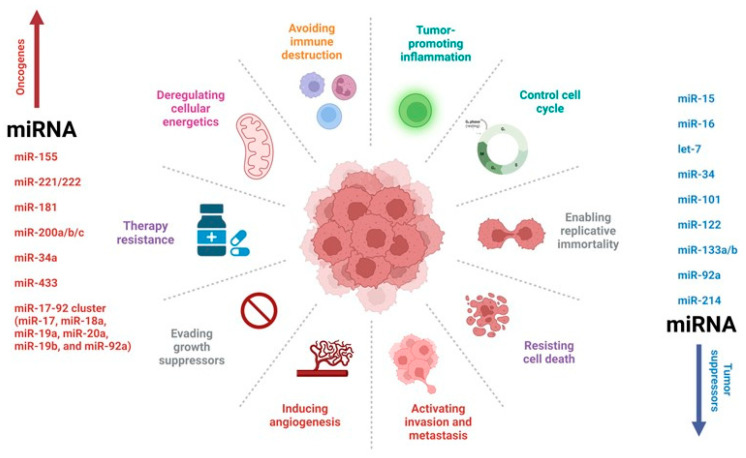
MicroRNA (miRNA) regulation of a tumor network. MiRNAs have been implicated in brain tumors, acting either as oncogenes or tumor suppressor genes. The most studied miRNAs to date have been shown to have multiple functions in tumor biology.

This interaction leads to a reduction in protein production either by obstructing the translation process or promoting the degradation of the target mRNA. An estimated 60% of human genes are directly regulated by miRNAs. Furthermore, certain miRNAs can associate with more than one mRNA target, at times within the context of the same signaling pathway. Conversely, specific mRNAs can encompass multiple distinct miRNA-binding regions within their 3′ UTR, adding layers of regulatory complexity [9]. Consequently, miRNAs serve as fine tuners of gene expression in response to abnormal cellular cues (Figure 2). For instance, C. Sippl and colleagues analyzed the change in expression levels of miR-21, miR-24, and miR-26a in tumor specimens of 104 patients with glioblastoma and 8 specimens of non-neoplastic brain tissue from the control group [10]. In their study, they demonstrated that miR-26a and miR-21 were significantly overexpressed in glioblastoma samples. In addition, high expression levels of miR-24 trended for the prolonged overall survival of glioblastoma patients; however, with high expression levels of miR-26a, a significantly prolonged progression-free survival was evident.

While most miRNAs are generated within cells themselves, a multitude of miRNAs, referred to as cell-free miRNAs, have been identified in various bodily fluids, such as blood or CSF [11]. The expression pattern of cell-free miRNAs experiences substantial alterations (deviations or disruptions) in various human ailments, including brain tumors [12]. These miRNAs display resistance to nucleases, making them attractive candidates as potential biomarkers for diagnosing, prognosticating, and monitoring therapeutic responses [12]. This study focuses on the possible utilization of cell-free miRNAs as biomarkers in the context of brain tumors.

## 2. Varieties and Mechanisms of Cell-Free miRNAs Secretion

At present, three secretion routes have been established: (1) passive release from damaged cells consequent to apoptosis or necrosis; (2) active release facilitated by extracellular vesicles (EVs), which comprise exosomes and microvesicles (MVs); and (3) active release mediated by the RNA-binding protein-dependent pathway (miRNA-Ago2 complex) (Figure 1) [11]. Several investigators communicated that, among the EVs identified in plasma or serum, MVs stand as the predominant subset [13]. MV contents display diversity, encapsulating lipids, mRNA, miRNAs, and proteins. Notably, MVs are equipped to selectively target recipient cells for transporting miRNAs, thereby instigating signal transduction cascades [14,15,16]. Exosomes, small membrane-bound vesicles stemming from endosomes with dimensions ranging from 30 to 100 nm, are emitted by various cell types, irrespective of their normal or pathological nature. Originally, exosomes were conceived as a cellular mechanism for eliminating extraneous “clutter,” such as obsolete proteins [15].

Nevertheless, contemporary research demonstrates that these vesicles extend beyond their initial classification as mere “waste containers”, occupying a significant role in intercellular communication. Exosomes, in this light, serve as conveyors of information (such as mRNA and viruses) and other materials (such as proteins and miRNAs) from one cell to another. Tumor cells assume a pivotal function in exosome generation. Exosomes originating from tumor cells possess the capacity to participate remotely in the establishment of pre-metastatic “niches” [17]. In addition to vesicular forms of cell-free miRNAs, apoptotic bodies with dimensions ranging from 1 to 4 nm can also be detected in biological fluids, harboring miRNAs within them [11].

MicroRNAs also have the potential to be secreted independently of vesicles. Approximately 90% of cell-free microRNAs exist in a non-vesicular state, specifically bound to Ago2 proteins [18]. Recent findings have confirmed that non-vesicular cell-free microRNAs, particularly those tethered to Ago2, are actively liberated from neurons [18]. Remarkably, these miRNAs were found to be released from a distinct cellular compartment: the distal end of the axon [11]. Furthermore, it has been reported that, in addition to the Ago2 protein, high-density lipoproteins (HDLs) are implicated in intercellular communication mechanisms and participate in the transport and conveyance of miRNAs. However, the origin of the lipoprotein fraction of cell-free miRNAs remains unexplored.

## 3. Advantages of Cell-Free miRNAs

**Figure 3:**
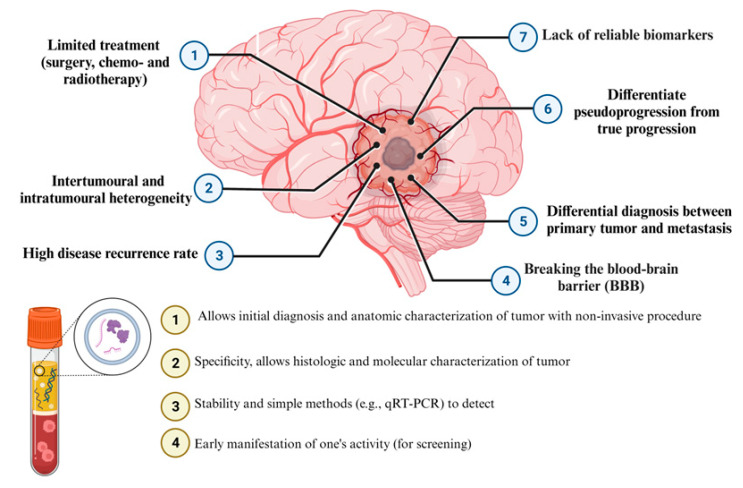
Diagnostic performance of liquid biopsy and detection cell-free microRNAs (miRNAs) in brain tumors.

The utilization of cell-free miRNAs as biomarkers holds immense potential in refining the management strategies for individuals with brain tumors, offering applications in various aspects, including early tumor detection, identification of treatment-resistant tumors, prompt recognition of tumor recurrence, monitoring responses to surgical interventions, chemotherapy, and radiation therapy, as well as optimizing the implementation of precision medicine methodologies (Figure 3).

### 3.1. Diagnostic Utility

One of the pivotal factors shaping the approach to treating patients afflicted with primary or metastatic tumors within the CNS is the accurate determination of the tumor type. Typically, tumor classification hinges on tissue analyses performed during surgical procedures, with subsequent therapeutic decisions contingent upon biopsy outcomes. Even among patients for whom surgical intervention is not feasible, such as those with CNS lymphoma, the current imperative remains obtaining tissue biopsies for precise diagnosis and subsequent treatment planning [19]. The potential to differentiate between CNS lymphoma and diffuse glioma through the assessment of liquid biopsy-derived biomarkers might obviate the need for tissue biopsies in specific scenarios. Furthermore, in the contemporary landscape of harnessing molecular parameters for the classification of CNS tumors, distinct cell-free miRNAs that bear prognostic significance as biomarkers can play a pivotal role in strategizing surgical interventions, making real-time intraoperative choices, and facilitating participation in clinical research endeavors.

### 3.2. Detection of Tumor Recurrence

Identifying the recurrence risk, whether in the short term or the long term, after the treatment of primary or metastatic brain tumors, presents a multifaceted conundrum. This complexity arises from the challenge of accurately distinguishing changes induced by the secondary effects of radiation therapy, known as tumor pseudo-progression, from actual tumor recurrence, termed true progression, using conventional visualization methods [20]. As experts often face difficulties in discerning between pseudo-progression and true recurrence via neuroimaging modalities, certain patients lacking genuine tumor recurrence may undergo potentially superfluous surgical interventions, such as repeated tissue biopsies, to confirm the resurgence of the tumor. Such surgical interventions bear the inherent risk of yielding a notably high rate of sampling errors.

Hypothesized is the correlation between the fluctuations in the expression levels of cell-free miRNAs and the tumor’s burden. In this scenario, a plausible conjecture is that the advancement of the tumor can lead to an elevation in the expression levels of specific cell-free miRNAs in patients with brain tumors. This can potentially serve as a discriminative factor in distinguishing between authentic progression and pseudo-progression [21]. The pivotal implication of such distinction lies in its potential to spare patients, who have undergone radiation therapy, from additional and avoidable surgical interventions.

### 3.3. Monitoring Treatment Response

Assessing the effectiveness of tumor treatment often proves challenging through conventional diagnostic modalities, including magnetic resonance imaging (MRI), perfusion MRI, computed tomography, MR spectroscopy, and positron emission tomography [22]. Despite the endeavors to identify biomarkers for monitoring treatment response, such as Ki-67 and p53 for CNS tumors, none have yet been able to establish both clinical sensitivity and specificity. The absence of biomarkers that are both sensitive and specific in gauging treatment responses among individuals with brain tumors serves as an impediment to the advancement of novel therapeutic interventions. Hence, future research endeavors must strategically focus on bridging this knowledge gap, systematically evaluating CSF or blood specimens for cell-free miRNAs and establishing correlations between the levels of miRNA expression and tumor volume as determined by visualization techniques.

## 4. Cerebrospinal Fluid or Blood?

When it comes to patients with brain tumors, blood (plasma/serum) emerges as a readily available biological fluid for assessing the expression patterns of cell-free microRNAs. The information gathered from a plasma analysis has proven valuable in diagnosing tumors, predicting their outcomes, gauging responses to therapy, tracking recurrences, and uncovering emerging treatment resistance across a spectrum of human cancers, including bladder, breast, colorectal, gastric-esophageal, hepatocellular, ovarian, pancreatic, and melanoma cancers [12,23,24]. Interestingly, the presence of cell-free miRNAs in plasma samples without evident circulating tumor cells (CTCs) hints at the potential of these miRNAs to offer insights into tumors independently of direct tumor cell presence in biofluids. Some studies have reported varying sensitivities of CTCs in glioblastoma patients’ blood, ranging from 21% to 39%. However, the blood–brain barrier (BBB) poses limitations, impacting the ideal use of blood for an accurate biomarker evaluation [25,26,27]. Moreover, blood might not be an optimal candidate for detecting metastatic brain tumors. Recent research has compellingly shown that the BBB can hinder CTCs or cell-free miRNAs from infiltrating the bloodstream. Nevertheless, just as observed in brain tumors, the compromised BBB’s integrity allows select molecules, including cell-free microRNAs, to permeate from the CNS into the blood circulation and potentially from the systemic circulation into the CSF. For example, a study led by S. Sorensen et al. [28] investigating the alterations in the cell-free miRNA profiles in the CSF and blood of ischemic stroke patients unveiled that half of the stroke patients exhibited elevated serum albumin quotient (Qalb) values. Interestingly, a parallel pattern emerged in three control patients with multiple sclerosis, indicating significant changes in the BBB’s functionality. This lends support to the notion that a substantial subset of patients from both groups encountered disruptions in the BBB, making it conceivable that some of the cell-free miRNAs detected in the CSF of these individuals originate from the bloodstream.

As previously explained, EVs are tiny particles enclosed by membranes that are re-leased from viable tumor cells, either by the merging of endosomes with the cell’s outer membrane (exosomes) or directly from the cell membrane itself (MVs) [11]. These EVs serve as carriers, connecting various sections of the tumor and its surrounding environment, as they are taken up by other tumor cells and normal cells alike [29]. It is crucial to highlight that EVs, which can be derived from both blood and CSF, serve as a rich reservoir of tumor-related molecules, encompassing DNA, miRNAs, mRNAs, proteins, lipids, and metabolites. Their structure safeguards these molecules from the actions of nucleases and proteases [30,31,32]. Given the prevalence of nucleases and proteases in blood, isolating miRNAs from EVs can result in higher RNA concentrations compared to non-vesicular forms of cell-free miRNAs found in whole blood, plasma, or serum [33]. Additionally, platelets can seclude the contents of EVs, and tumor-associated miRNAs have been identified within platelets from individuals with glioblastoma [34]. This additional information provides insight into the possible origin of cell-free miRNAs, in particular, their tumor cells, as well as understanding about oncogenesis and the role of miRNAs in the regulation of several oncogenes, such as epidermal growth factor receptor variant III (EGFRvIII) [34,35,36].

In contrast to blood, CSF is in direct contact with the CNS and is proven to be a suitable reservoir of biomarkers for brain tumors. Furthermore, obtaining CSF is a simpler and safer process than procuring tumor tissue through biopsies. In a study conducted by Y. Yagi et al. [37], alterations in the expression of exosomal miRNAs within the CSF of healthy individuals were showcased using next-generation sequencing (NGS). Their findings revealed that the expression pattern of cell-free miRNAs in CSF is predominantly linked with the exosomal fraction. Furthermore, the presence of exosomal miR-1911, which is distinctly overexpressed in healthy brain tissue, was identified in CSF but not in blood serum. This conclusion was reaffirmed through digital polymerase chain reaction tests conducted on samples from three healthy donors. This significant discovery suggests that exosomal miRNAs within CSF can also serve as indicators of CNS pathology. Intriguingly, the previously documented expression of miR-1911 was suppressed in glioma tissue specimens [38]. This specific miRNA can potentially function as a tumor suppressor, and its deactivation might influence the transformation of glial cells into malignancy. All these observations underscore the necessity for further research that regards this microRNA as a plausible biomarker within CSF for gliomas.

As has been documented, miRNAs are present within HDLs [11]. The available data suggest that the cholesterol concentration in CSF is lower than 0.5% of the concentration found in blood serum, indicating that the relatively diminished levels of cell-free miRNAs in CSF supernatant can be associated with the reduced presence of HDL particles [39]. Additionally, the HDL particles in CSF diverge from those in blood plasma in that the predominant component in the former scenario is apolipoprotein E, as opposed to apolipoprotein A1 (ApoA-I) [40]. Given that ApoA-I plays a role in the transport of miRNAs, this contrast between CSF HDL molecules and plasma HDL molecules can potentially influence their interaction with miRNAs [41]. Despite the origin of miRNA-Ago2 in CSF remaining unknown, the quantity of cell-free miRNAs linked with the Ago2 complex in CSF is comparatively lower than in the bloodstream.

The expression pattern of cell-free miRNAs in CSF might diverge from that observed in blood (serum or plasma); however, miRNAs exhibiting elevated expression levels in both CSF and blood signify the regulation of the same set of genes and share numerous common signaling pathways associated with tumor-related neuroinflammation and the disruption of BBB.

## 5. Cell-Free miRNAs as Diagnostic and Prognostic Biomarkers

The exploration and development of effective therapeutic treatments for diverse human diseases have firmly established phases and processes, such as those observed in clinical trial stages. However, adapting these identical phases and processes for biomarker research, particularly for the early diagnosis and prognosis of cancerous conditions, presents a challenge. Toward the latter part of the 20th century, the National Cancer Institute (NCI) and other research collectives commenced constructing a systematic framework for the recognition and substantiation of biomarkers. Due to the profound significance of early tumor identification in public health, the NCI established the Early Detection Research Network (EDRN) research framework. This endeavor aimed to identify and validate tumor biomarkers, while simultaneously designing a methodical approach to uncover and substantiate biomarkers for screening and diagnostic purposes [42]. The EDRN plan encompassed a five-phase algorithm for investigating and ascertaining effective biomarkers targeting the early detection of tumors and pre-tumor states. This sequential technique garnered substantial acceptance within the biomarker research community. In recent years, remarkable progress has been achieved in the application of biomarkers to diagnose brain tumors in standard medical practice, such as the methylation of the O6-methylguanine-DNA methyltransferase gene promoter in gliomas [43].

Among the extensively studied biomarkers are cell-free miRNAs. While not yet integrated into clinical practice, advancements in this realm underscore the potential of cell-free miRNAs as pivotal tools for diagnosing and predicting brain tumors. Furthermore, there is the prospect that these miRNAs might even supplant certain stages in contemporary diagnostic procedures [44,45,46,47,48,49,50,51,52,53,54]. To exemplify, the replacement of conventional tissue biopsies with the so-called “liquid biopsy” holds the potential to obviate diagnostic surgical interventions, thereby reducing the risk of potential complications. A case in point, J. Wang et al. [44] revealed a substantial elevation in the expression level of cell-free miR-214 in the bloodstream of patients with grade-I and -II malignancy gliomas, in comparison to the control group. However, patients afflicted with grade-I gliomas exhibited a more pronounced elevation in cell-free miR-214 expression than their grade-II counterparts. Furthermore, the increased presence of cell-free miR-214 in the bloodstream exhibited a significant correlation with the absence of isocitrate dehydrogenase 1 and 2 (IDH 1/2) gene mutation and the presence of an unmethylated promoter in the methylguanine methyltransferase (MGMT) gene. Additionally, a receiver operating characteristic (ROC) analysis was undertaken to gauge the diagnostic effectiveness of cell-free miR-214, revealing an exceptionally high area under the curve (AUC) of 0.885 (95% CI 0.833—0.926) when comparing patients with grade-I and -II malignancy gliomas against the control group. The authors also deduced that the heightened expression of cell-free miR-214 in glioma patients was associated with a bleaker prognosis. Furthermore, miR-214 can function independently as a prognostic predictor of overall survival in gliomas, particularly in more severe malignancies (grade-II gliomas).

Utilizing the NGS technique, the research has unveiled distinct variations in the expression of 169 exosomal miRNAs within the blood serum of individuals afflicted with growth hormone (GH)-secreting pituitary adenomas when compared to the control cohort [48]. Among these 169 miRNAs, the manifestation of cell-free miR-423-5p exhibited a pronounced reduction in the serum of the study subjects in contrast to the control group, as substantiated by the analysis conducted via miRSCan Panel Chip qPCR. Moreover, the investigation highlighted that the upregulation of miR-423-5p prompted apoptosis in tumor cells, curtailed their proliferation and migratory capabilities, and mitigated the release of GH. These findings suggest a prospective involvement of miR-423-5p in the pathogenesis of GH-secreting pituitary adenomas. The presence of exosomal miR-423-5p within blood serum can potentially function as a non-invasive biomarker; however, this proposition necessitates further in-depth exploration.

The scientific literature indicates that miR-330 operates as a tumor suppressor in prostate cancer [55,56], and a high miR-330 expression can repress the propagation of colorectal cancer cells in vivo [57]. Nevertheless, the precise roles and underlying molecular mechanisms of miR-330 in the regulation of lung cancer remain enigmatic. Through the utilization of quantitative real-time polymerase chain reaction (qRT-PCR), Jiang and colleagues [53] delved into the ramifications of miR-330 in the context of radioresistance and brain metastasis within lung cancer, alongside its potential utility as a cell-free serum biomarker. The researchers revealed that the expression of cell-free miR-330 was diminished in radioresistant patients afflicted with brain metastasis, and this diminished expression was found to be linked to a reduced average survival duration. These results intimate that the attenuation of miR-330 expression might have been influenced by the effects of radiotherapy and exhibit a correlation with unfavorable prognoses in individuals with metastatic conditions. Thus, the existence of cell-free miR-330 might serve as an innovative predictor of radiation responsiveness and prognostic outlook for patients enduring lung cancer that has metastasized to the brain.

Sippl and colleagues [58] showed a significant overexpression of miR-181d in the tumor tissue and plasma of glioblastoma patients compared with healthy individuals. Even if the majority of prognostic and predictive GB biomarkers are currently developed using tumor samples obtained through surgical interventions [59,60], the AUC values indicate that the two groups may be distinguished by the expression analysis of miR-181d. In addition, the authors demonstrated that The Cancer Genome Atlas analysis revealed 8 potential protein targets to be regulated by miR-181d.

In Table 2 and Table 3, we summarize some research articles where the authors studied cell-free miRNAs as non-invasive biomarkers for the diagnosis and prognosis of brain tumors [44,45,61,62,63,64,65,66,67,68,69,70,71,72].

## 6. Future Perspectives and Limitations

Despite numerous efforts to enhance the outcomes for individuals with brain tumors, the survival rates have not shown a significant improvement. There is an existing need to integrate the progress made in tumor biology into clinical approaches for brain tumors, given that the recent clinical trials investigating targeted therapies have not yielded effective results for the broader population of registered brain tumor patients. Various distinct characteristics of tumors in this location can contribute to these challenges. Primarily, brain tumors, particularly gliomas, possess an intricate biology that complicates the comprehension of intratumoral heterogeneity and the dynamics of clonal and subclonal tumor structures during the progression of the disease, hindering the prevention of tumor adaptation and drug resistance [72,73]. Furthermore, there is an absence at present of non-invasive tools that can facilitate the more accurate monitoring of tumor progression [74,75]. Additionally, the treatment of CNS tumors presents a unique obstacle due to the difficulties associated with delivering drugs across the BBB, as also to other factors, such as radio-chemotherapy, the pre-operative status, and systemic immunity [76,77,78]. Cell-free miRNAs have the potential to assist medical professionals in addressing some of these challenges.

From the standpoint of an oncologist or neurosurgeon, cell-free miRNAs offer a significant advantage by supplying real-time molecular insights without necessitating invasive interventions on the brain, particularly during tumor recurrence. The integration of information derived from neuroimaging techniques, such as brain MRI data, alongside clinical evaluations, and the measurements of cell-free miRNA expression levels in bodily fluids, will undoubtedly play a critical role in the future for accurately characterizing specific tumor processes (e.g., distinguishing between tumor progression and pseudo-progression) within the context of clinical trials or routine patient care.

A central query within the domain of cell-free miRNA research as biomarkers revolves around the selection of the primary biological fluid (blood or cerebrospinal fluid), demanding a resolution to fully harness the potential of cell-free miRNAs in diagnosing, prognosticating, and formulating therapies for brain tumors. Venipuncture emerges as a simpler and less intrusive method compared to lumbar puncture, rendering the exploration and identification of blood-based biomarkers particularly enticing. Nevertheless, owing to the isolation of tumor tissue from the blood supply of the BBB, coupled with the direct interaction of CSF with tumor tissue, a conjecture arises that cell-free microRNAs derived from CSF can offer more dependable biomarkers for brain tumors. The optimization of using cell-free miRNAs as biomarkers for brain tumors hinges on the refinement of expression profiling techniques, ensuring they attain ample sensitivity and specificity for a quantitative analysis with minute sample volumes. Despite the notable progress in technology for profiling cell-free miRNA expression, their utility as biomarkers confronts specific constraints. Notable among these constraints is the absence of standardized protocols for handling and storing samples within clinical contexts. Moreover, a limited understanding of the environmental factors capable of influencing cell-free miRNA expression in individuals with brain tumors might curtail their clinical applicability (Table 4) [79,80,81,82,83,84,85,86,87,88,89,90,91].

## 7. Conclusions

MicroRNAs, small non-coding RNA molecules, wield significant control over a vast portion of the human genome, intricately influencing an array of cellular processes. Their regulatory role extends to approximately one-third of the human genome, orchestrating processes, such as tumor cell proliferation, programmed cell death (apoptosis), cell movement (migration), invasion of neighboring tissues, and the formation of new blood vessels (angiogenesis). By targeting a multitude of genes, miRNAs play a pivotal role in shaping the behavior and characteristics of tumor cells. Within the context of brain tumors, these microRNAs assume an even more critical role. They actively participate in modulating the degree of malignancy exhibited by brain tumors and influencing the process of cellular differentiation. This intricate involvement suggests that the dysregulation of specific microRNAs can be a valuable indicator of clinical prognosis. As researchers delve deeper, it becomes evident that the expression profiles of these miRNAs hold immense potential for diagnostic and prognostic applications. A compelling thread of evidence emerges from numerous studies, advocating for the utilization of cell-free microRNAs in human biological fluids for diagnostic and prognostic analyses. Both cerebrospinal fluid (CSF) and blood emerge as promising sources of these cell-free microRNAs. This holds particularly true for various CNS disorders, including the formidable challenge of brain tumors. Yet, the potential of miRNAs extends beyond diagnosis and prognosis. By scrutinizing the expression patterns of cell-free miRNAs, clinicians might gain an insight into the recurrence of brain tumors, thereby tailoring their therapeutic strategies more effectively. Additionally, the power of these molecules can enable ongoing surveillance, providing a real-time understanding of treatment efficacy, especially concerning chemotherapy and radiotherapy. The uniqueness of cell-free miRNAs within cerebrospinal fluid and blood presents a tantalizing perspective. This uniqueness is driven by the inherent differences in these biological fluids. Cerebrospinal fluid, being proximate to brain tissue, has the potential to reflect localized events and disruptions within the delicate neural environment. On the other hand, blood, cell-free throughout the body, carries a broader systemic perspective. This divergence offers an opportunity to glean insights into both localized and systemic responses to brain tumor pathophysiology. Looking ahead, as technology refines and expands our understanding of these miRNA signatures, we can envision a future where specific panels of cell-free miRNAs not only aid in distinguishing brain tumors from other central nervous system conditions but also offer a dynamic window into the intricate landscape of brain tumor progression, responses to therapy, and overall patient management. This endeavor holds the promise of translating complex molecular insights into tangible improvements in patient outcomes, starting a new era of precision medicine for brain tumor patients.

## Figures and Tables

**Table 1:** Advantages and disadvantages of employing liquid biopsies for the detection and monitoring of brain tumors.

Source	Factor	Advantages	Disadvantages	References
CSF, blood, urine	miRNA	**Specificity +/−** There is a chance for the convenient and precise tracking of therapy effectiveness, and even the possibility of using it for initial tumor diagnosis, contingent on the choice of appropriate markers or panels	**Sensitivity − − −** Lacks distinctive tumor-specific sequences, requires a comparison with normal references, lacks standardization	[4,5]
CSF, blood, neurosurgical fluid (including DNA, RNA, miRNA)	EV	**Specificity + + +** CSF is preferable to blood because it contains fewer background signals from white blood cells	**Sensitivity −** Signals originating from regular cells (leukocytes, for instance), as seen in blood	[5,6]
CSF, blood	Cell-free nucleic acid (DNA, RNA)	**Specificity + + +** Molecular assessment using established techniques, simpler to collect compared to CTCs, rapid and convenient tracking of tumor progression and treatment reaction, might constitute a massive portion of the tumor and surpass localized tissue biopsies in value	**Sensitivity −** Might not accurately depict the entirety of the tumor, lacks a definitive established standard, contingent on the tumor’s proximity to CSF	[4,5,6]
CSF, blood	CTC	**Specificity + + +** Utilizing molecular diagnosis enables swift and effortless tracking of both tumor progression and treatment responsiveness. It has the potential to portray a pertinent portion of the tumor and could outperform conventional local tissue biopsies	**Sensitivity − − −** Highly uncommon, challenging to isolate, lacking established norms, might not accurately reflect the entirety of the tumor, necessitates further experimental investigations	[5,7,8]

CSF, cerebral spinal fluid; EV, extracellular vesicle; CTCs, circulating tumor cells; microRNA (miRNA).

**Table 2:** Cell-free microRNAs (miRNAs) as diagnostic biomarkers in brain tumors patients.

Tumor	Number of Patients, n	miRNA	Regulation	Biofluid	AUC	Sensitivity, %	Specificity, %	Important Find	Reference
Glioma (WHO grades II and IV)	47 and 44	miR-320e, miR-223, miR-21, miR-23a	Up	Serum	99.8	100.0	97.8	Confirming the diagnosis of pseudo-progression	[61]
Glioma (WHO grades II and III–IV)	11 and 11	miR-125b	Down	Serum	0.868 and 0.959	81.82 and 90.91	75.76 and 87.88	Biomarker development, especially for WHO grade-II–IV gliomas	[62]
Glioblastoma	13	EV miR-21	Down	CSF	0.91	87.0	93.0	Glioblastoma cells actively secrete EVs containing miR-21	[63]
PCNSL	56	miR-21	Up	Serum and CSF	0.930	-	-	Correlation analysis demonstrated that serum miR-21 might reflect its companions in CSF	[64]
JPA	3	miR-26a-5p	Up	Serum	0.751	-	-	Correlated strongly in JPA patients within both the serum and tumor tissue samples	[65]
SCNSL and PCNSL	61 and 14	miR-30c	Up	CSF	0.86	90.9	85.5	miR-30c may facilitate lymphoma cells to engraft into CNS by the interaction with the CELSR3 gene that controls the function of ependymal cilia and, thus, affects the circulation of CSF	[66]
Glioma (WHO grades II and III–IV)	2 and 8	miR-15b	Up	CSF	0.96	90.0	94.9	Biomarker development, especially for WHO gradeII–IV glioma	[67]
Meningioma (WHO grades II–III)	40	miR-197 and miR-219a, miR-34a, miR-224 and miR-375	Up and down	Serum	0.79	-	-	miR-197, miR-34a, miR-375 for grade I, and miR-375 for grade II	[68]
Brain metastasis related to advanced breast cancer	51	miR-4428 and miR-4480	Up	Serum	0.779 and 0.781	-	-	Specific for brain metastasis (breast cancer)	[69]
Glioma (WHO grades I–II and III–IV)	38 and 62	miR-214	Up	Serum	0.885	90.00	71.00	Potential minimally invasive biomarker for tumor stratification, early detection	[44]

EVs, extracellular vesicles; PCNSL, primary central nervous system lymphoma; CSF, cerebral spinal fluid; JPA, juvenile pilocytic astrocytoma; SCNSL, secondary central nervous system lymphoma; AUC, area under ROC curve; EV, extracellular vesicle; CELSR3, cadherin EGF LAG seven-pass G-type receptor 3; AUC is considered diagnostically significant for the biomarker; -, not mentioned in the article.

**Table 3:** Cell-free microRNAs (miRNAs) as prognostic biomarkers in brain tumors patients.

Tumor	Number of Patients, n	miRNA	Regulation	Biofluid	Important Find	Reference
PCNSL	56	miR-21	Up	Serum and CSF	miR-21 as an independent and powerful predictor of overall survival	[64]
Glioma (WHO grades I–II and III–IV)	38 and 62	miR-214	Up	Serum	miR-214 as an independent and powerful predictor of overall survival	[44]
Glioblastoma	66	Exosomal miR-301a	Up	Serum	Exosomal miR-301a as an independent and powerful predictor of overall survival	[70]
Glioma (WHO grades III–IV)	64	miR-205	Down	Serum	miR-205 as an independent and powerful predictor of overall survival	[45]
Meningioma (WHO grades I–III)	230	miR-106a-5p, miR-219-5p, miR-375, miR-409-3p, miR-197-3p, and miR-224-5p	Up and down	Serum	Serum 6-miRNA as an independent and powerful predictor of overall survival	[45]
Glioblastoma, breast cancer metastasis to brain and leptomeningeal metastasis, lung cancer metastasis to brain and leptomeningeal metastasis	19, 16, 26, 28, and 4	miR-10b, miR-21, and miR-200 family	Up	CSF	miR-10b, miR-21, and miR-200 family as an independent and powerful predictor of overall survival of primary and metastatic brain tumors	[71]

Kaplan–Meier curve analysis and multivariable Cox regression as an independent and powerful predictor of overall survival; comb., combined; CSF, cerebral spinal fluid; PCNSL, primary central nervous system lymphoma.

**Table 4:** MiRNAs exhibit altered expression patterns in brain metastases when contrasted with the primary tumor. Altered miRNA profiles have been detected in metastatic brain tumor cells in comparison to their corresponding primary tumors.

MiRNA	Primary Tumor	Regulation	Potential Target	Reference
miR-19a	Breast	Down	3′-UTR of tissue factor transcript	[79]
miR-29c	Breast and melanoma	Down	Induced myeloid leukemia cell differentiation protein MCL1	[80]
miR-31	Colon	Down	p53	[81]
miR-200	Breast and lung	Up	E-cadherin transcriptional repressors ZEB1 and ZEB2	[82]
miR-210	Breast and melanoma	Up	PTP1b and HIF-1α	[83]
miR-1258	Breast	Down	Heparanase	[84]
miR-7	Breast	Down	*KLF4* gene	[85]
miR-145	Lung adenocarcinoma	Down	3′-UTR of the JAM-A and fascin	[86]
miR-328	NSCLC	Up	*PRKCA* gene	[87]
miR-378	NSCLC	Up	MMP-7, MMP-9, and VEGF	[88]
miR-146-a	Breast	Down	B-catenin and hnRNPC	[89]
miR-768-3p	Lung and breast	Down	K-RAS	[90]
miR-1, miR-145, miR-146a, miR-143, miR-10b, miR-22	Colon	Up	Multiple genes related to apoptosis and oncogenesis	[91]

PTB1b, protein tyrosine phosphatase-1B; HIF-1α, hypoxia-inducible factor 1-α; NSCLC, non-small cell lung cancer; MMP, matrix metalloproteinase; VEGF, vascular endothelial growth factor; ZEB1/2, zinc finger E-Box binding homeobox 1/2; KLF4, Kruppel-like factor 4; JAM-A, junctional adhesion molecule-A; hnRNPC, heterogeneous nuclear ribonucleoprotein C; K-RAS, Kirsten rat sarcoma 2 viral oncogene homolog.
